# What the Silent Retina Tells You: Serous Retinal Detachment in Waldenström’s Macroglobulinemia

**DOI:** 10.1097/HS9.0000000000000527

**Published:** 2021-01-20

**Authors:** Karima Amaador, Roselie Diederen, Jeroen Coumou, Josephine Vos

**Affiliations:** 1 Department of Hematology, Amsterdam UMC, University of Amsterdam, Amsterdam, The Netherlands; 2 Department of Ophthalmology, Amsterdam UMC, University of Amsterdam, Amsterdam, The Netherlands; 3 Department of Internal Medicine, Tergooi Ziekenhuis, Hilversum, The Netherlands; 4 Lymphoma and Myeloma Center Amsterdam (LYMMCARE), The Netherlands.

A 48-year-old woman presented with blurred vision, left more than right, at the ophthalmology clinic. The visual acuity was 20/22 (right eye) and 20/28 (left eye). Funduscopic examination led to a presumptive diagnosis of hypertensive retinopathy, and she was treated with antihypertensive medication. Six months later, a repeated and more extensive ophthalmologic examination demonstrated normalization of her visual acuity to 20/20 in the right eye but further deterioration to 20/50 in the left eye despite normalization of her blood pressure. Optical coherence tomography (OCT) revealed marked subretinal fluid accumulation near the inferior macula, most pronounced in the left eye (Figure 1A, B). On fundus examination, tortuous vessels in the left eye were seen, consistent with hyperviscosity syndrome (HVS) (Figure 1E, F). On fluorescein angiography (FAG), there was no leakage over the area of neurosensory detachment (Figure 1G, H). A diagnosis of serous retinal detachment was made, with concomitant hyperviscosity-related damage. The ophthalmologist referred her to the hematology clinic. A diagnosis of Waldenström’s macroglobulinemia (WM) was made based on immunoglobulin M (IgM) paraproteinemia with a total IgM level of 9818 mg/dL and 50% bone marrow infiltration with Lymphoplasmacytic Lymphoma cells (MYD88 L265P, CXCR4 T318fs mutated).

**Figure 1.**
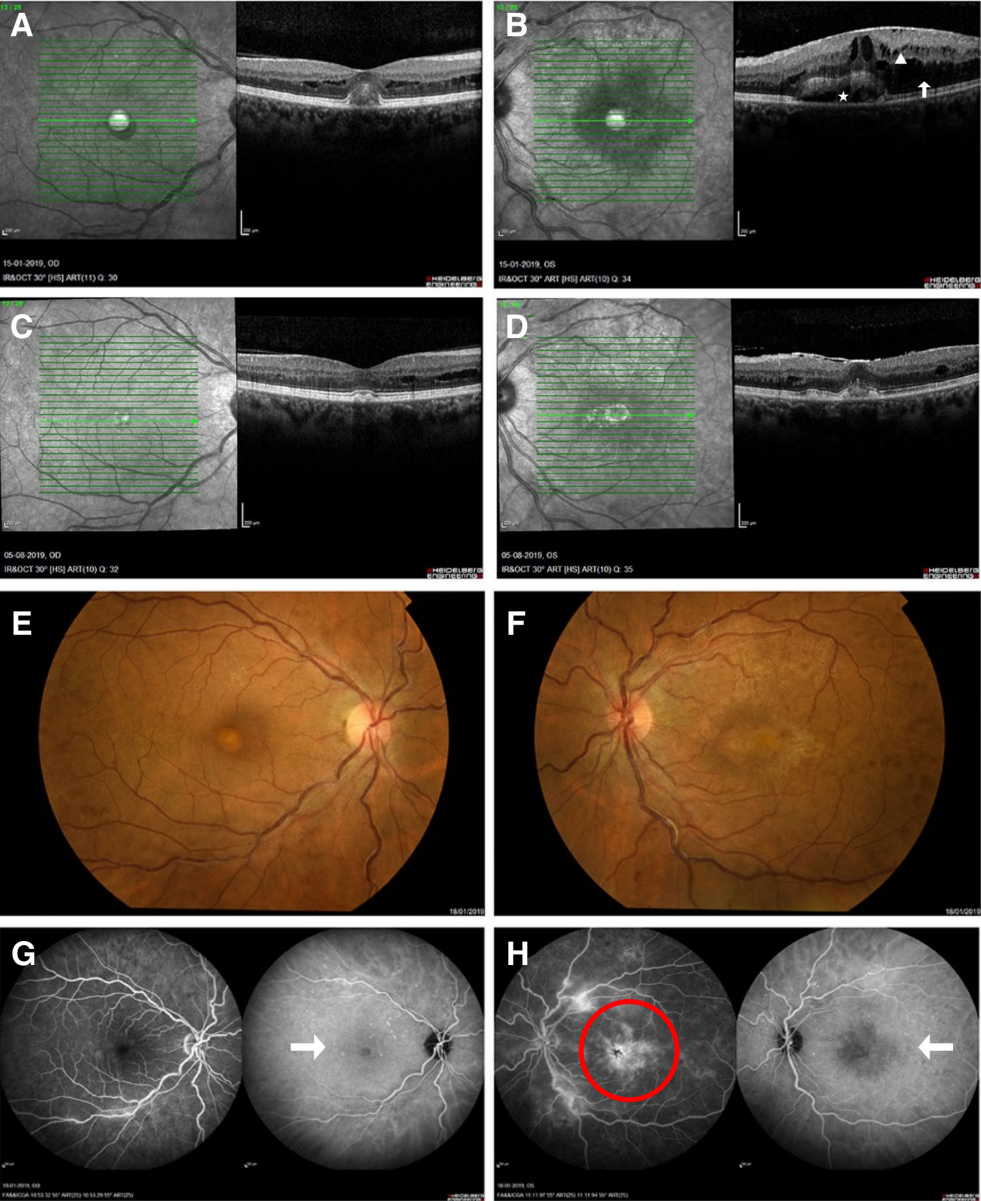
**Imaging of a 48-year-old woman with Waldenström’s macrogobulinemia.** Optical coherence tomography on initial presentation of the right (A) and left eye (B) demonstrates intraretinal (△) and subretinal fluid (↑) with cystoid edema surrounding the area of detachment with RPE elevation (⋆) left worse than right. Optical coherence tomography 7 months after presentation of the right (C) and left (D) eyes demonstrating significant decrease of intraretinal and subretinal fluid in the left eye. The intraretinal fluid in the right eye demonstrated subtle decrease. Fundus photographs showing dilated tortuous veins, scattered intraretinal hemorrhages, and mild cystoid macular edema (E and F). Fluorescein angiography shows numerous microaneurysms most prominent in the midperiphery of both eyes, foveal edema (red circle), and no leakage in the macula of both eyes (↑) (G and H). RPE = retinal pigment epithelium.

She immediately started plasmapheresis as well as WM therapy. She had no response (IgM 8620 mg/dL) and minor response (IgM 7100 mg/dL) to first- (cyclophosphamide/dexamethasone) and second-line treatment (ixazomib/dexamethasone given as trial medication), respectively. Her visual complaints worsened as serum IgM increased again due to WM relapse, and she was referred to our center. Third line treatment with ibrutinib was initiated. In addition, she was started on monthly intravitreal injections of bevacizumab (an anti-vascular endothelial growth factor [VEGF] agent). This resulted in a slight decrease in IgM, subtle improvement of her vision to 20/32, and decrease of intraretinal fluid on OCT of her left eye.

However, ibrutinib had to be discontinued after 12 weeks due to ibrutinib-induced hepatotoxicity (Common Terminology Criteria for Adverse Events [CTCAE] grade 3). She progressed as her hemoglobin level decreased and IgM level increased to 7870 mg/dL. She was started on prolonged weekly plasmapheresis, which led to a durable lowering of the IgM level at around 4000 mg/dL. Since there was no further ocular improvement after 3 bevacizumab injections, she was converted to monthly aflibercept (a second-generation VEGF inhibitor). Simultaneously, 6 cycles of bendamustine, rituximab combined with bortezomib/dexamethasone were given and the IgM eventually decreased to 900 mg/dL. Her vision almost normalized to 20/25 in the left eye (Figure 2). A repeat OCT revealed impressive decrease in intraretinal and subretinal fluid in the left eye (Figure 1C, D). An OCT performed 2 months later (not shown) demonstrates complete resolution of subretinal fluid. Over time, the subretinal edema dissolved much slower than the funduscopic abnormalities consistent with HVS.

About 14% of WM patients present with HVS at the time of diagnosis and this is a well-known presentation of the disease. Typical hyperviscosity-related abnormalities seen on fundoscopy include retinal hemorrhages, microaneurysms, venous occlusions, tortuous veins, and papilledema.^1^ However, a rare vision-threatening complication in WM is serous macular detachment (SMD), as illustrated by this case.

The diagnosis can be made with OCT and FAG. The crucial feature of SMD, as seen in WM patients, is subretinal edema with the absence of leakage on FAG, also termed “silent macula.” Only 18 cases of “silent” SMD have been published, 15 in WM, 2 in Multiple myeloma, and 1 in benign polyclonal gammopathy.^2–14^ All other varieties of SMD (such as common age-related macular degeneration, diabetic or hypertensive retinopathy, infectious/inflammatory choroidopathies) demonstrate angiographic leakage.^4^ Therefore, SMD without angiographic leakage is virtually pathognomonic for WM (and seldomly other gammopathies) and should prompt evaluation for gammaglobulin levels.^4,10^

The pathophysiology is not fully elucidated. In the physiological situation, there is a barrier formed by retinal pigment epithelium (RPE) that prevents fluid from entering the subretinal space (the blood-retina barrier). Local ischemia related to high IgM levels may lead to outer retinal defects. These may cause leakage of IgM to the subretinal compartment, creating an increased osmotic gradient, which results in edema.^7,12,15^ In addition, the RPE’s pump mechanism can be compromised by the high IgM levels resulting in further subretinal fluid accumulation.^13^ These mechanisms strongly suggest that high IgM levels play a crucial role in the pathophysiology of SMD. This is reflected in the literature by a median IgM level of 5300 mg/dL in the reported cases of WM patients with SMD.^3–5,7,12–14^

While some classify SMD as a separate entity, one might also classify it as an atypical variant of HVS.^12^ Indeed, SMD can occur with and without other signs of HVS. Two published cases with “silent” SMD in the literature did not have concurrent hyperviscosity-related funduscopic abnormalities, and we also treated a similar case.^8,14^

There is no consensus on the preferred treatment approach of this rare manifestation of WM. Since SMD is a vision-threatening complication, we suggest considering it as a hematological emergency (like HVS), prompting plasmapheresis and the initiation of a rapidly acting WM treatment.

In the 15 reported cases, the WM treatment regimens were very heterogeneous and ocular responses were variable; many cases still had persistent sub- and intraretinal fluid without improvement of visual acuity.^3–5,7,9,12,14,16^

Intravitreal anti-VEGF injections have been applied in this context because of their known efficacy in other forms of subretinal fluid and/or macular edema. The role of local therapy with intravitreal anti-VEGF injections remains unclear as these have shown varying results in the literature.^9,11,12^ In 2 case reports, there was improvement in visual acuity and resolution of subretinal fluid after treatment with intravitreal bevacizumab; however, this was applied concurrently with systemic therapy and plasmapheresis.^9,11^ The effect of VEGF injections versus the prolonged lowering of IgM levels cannot be distinguished from one another. It is our impression that the subretinal edema responds slowly since it takes time to be reabsorbed, in contrast to the HVS associated changes that are rather rapidly reversed. We feel that rapid and durable suppression of IgM levels is probably key to ocular improvement, and intravitreal anti-VEGF injections might aid in this process by supporting optimal retinal circulation.

Summarizing, SMD should be recognized as a rare but vision-threatening manifestation of WM and should therefore be treated as a hematological emergency. The diagnosis can be confirmed by the presence of subretinal edema “without” leakage on FAG, and this should prompt hematological evaluation including IgM gammaglobulin levels. The therapeutic approach should consist of immediate lowering of the IgM by initiation of plasmapheresis and a fast acting WM treatment. Intravitreal injections of anti-VEGF drugs like bevacizumab or aflibercept can be considered as supportive. The goal of these combined measures would be to restore the retinal circulation, protect the retina from hypoxia, and ultimately resolve the edema and the visual impairment.

**Figure 2.**
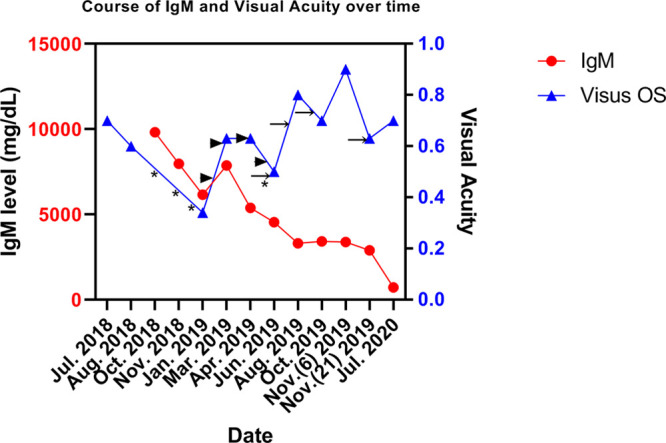
**The course** **of IgM levels and visual acuity over time in our patient.** The arrowheads/arrows indicate the time points at which intravitreal anti-VEGF drugs were administered (arrowheads: bevacizumab; arrows: aflibercept). The asterisks indicate start of treatment in the following order: cyclophosphamide/dexamethasone, Ixazomib/dexamethasone, ibrutinib/dexamethasone, and bendamustine/rituximab combined with bortezomib/dexamethasone. IgM = immunoglobulin M; VEGF = vascular endothelial growth factor; OS = oculus sinister.

## Disclosures

The authors have no conflicts of interest to disclose.
